# Effects of the free healthcare policy on maternal and child health in Burkina Faso: a nationwide evaluation using interrupted time-series analysis

**DOI:** 10.1186/s13561-023-00443-w

**Published:** 2023-05-05

**Authors:** Patrick Gueswendé Ilboudo, Alain Siri

**Affiliations:** 1 African Population and Health Research Center, Nairobi, Kenya; 2 Secrétariat Permanent du Plan National de Développement Economique et Social (SP/PNDES), Ouagadougou, Burkina Faso; 3 Institut des Sciences des Sociétés, Ouagadougou, Burkina Faso

**Keywords:** User fee removal policy, Effects, Costs, Interrupted time-series regression, Burkina Faso

## Abstract

**Background:**

Burkina Faso has recently instituted a free healthcare policy for women and children under five. This comprehensive study examined the effects of this policy on the use of services, health outcomes, and removal of costs.

**Methods:**

Interrupted time-series regressions were used to investigate the effects of the policy on the use of health services and health outcomes. In addition, an analysis of household expenditures was conducted to assess the effects of spending on delivery, care for children, and other exempted (antenatal, postnatal, etc.) services on household expenditures.

**Results:**

The findings show that the user fee removal policy significantly increased the use of healthcare facilities for child consultations and reduced mortality from severe malaria in children under the age of five years. It also has increased the use of health facilities for assisted deliveries, complicated deliveries, and second antenatal visits, and reduced cesarean deliveries and intrahospital infant mortality, although not significantly. While the policy has failed to remove all costs, it decreased household costs to some extent. In addition, the effects of the user fee removal policy seemed higher in districts with non-compromised security for most of the studied indicators.

**Conclusions:**

Given the positive effects, the findings of this investigation support the pursuit of implementing the free healthcare policy for maternal and child care.

**Supplementary Information:**

The online version contains supplementary material available at 10.1186/s13561-023-00443-w.

## Introduction

Over the last two decades, the improvement of maternal health and the health of children under five has been a critical priority for the international community through Millennium Development Goals 4 and 5 [1]. Despite this, the burden of maternal and child mortality has remained extremely high in many countries, particularly in sub-Saharan Africa. Several barriers, including delayed access to emergency obstetric care, especially cesarean sections, and widespread poverty, are significant obstacles to improving health outcomes for mothers and children. In settings with pervasive poverty, user charges have been an impediment for many households, preventing women from seeking qualified care during pregnancy or delivery, even in the event of complications [2]. Those who access care experience substantial difficulties paying for hospital fees and often resort to coping strategies such as selling assets, borrowing from friends or family members or accruing new debts to meet the costs [3]. These may lead to long-term negative consequences [4, 5].

Several sub-Saharan countries, including Burkina Faso, Burundi, Gabon, Ghana, Kenya, Liberia, Nigeria, Senegal and Uganda, have substantially or entirely removed user fees for maternal care and children under five [6–9]. The rationale behind such policies is the broad recognition that user fees constitute a significant financial barrier to accessing healthcare, especially for vulnerable groups such as women, children and the poorest segment of the population [8, 9]. User fee removal policies aim to facilitate access to care by lowering/alleviating financial barriers at the point of care. Removal of the user fees, in turn, will contribute to increased use of qualified services and, ultimately, to improved health outcomes [10].

Impacts of user fee removals still need to be well documented in Burkina Faso. Before the introduction of the free healthcare policy for maternal and child health in Burkina Faso, only a few studies, using interrupted time series, evaluated isolated experiments to reduce or eliminate the cost of child healthcare based on limited temporal and/or geographical coverage in the North or Sahel regions [11, 12]. A more recent study has examined the cross effects of performance-based financing and user fee removal policies in selected districts in Burkina Faso [13]. They showed that removing user fees had increased the use of health services for consultations in children under five. None of these previous studies has, at the national level, thoroughly investigated the effects of the free healthcare policy on the use of healthcare services and outcomes. In addition, they have yet to investigate the effectiveness of the policy in eliminating costs. This study investigates the effects of user fee removal on health services usage and outcomes at the country level. It also analyzes the effectiveness of the policy in removing household costs.

## Methods

### Study setting and health policy initiatives

Burkina Faso is a low-income landlocked country in West Africa with a total population of 20,505,155 as of 2021 [14]. Women of reproductive age and children constitute 17.43% and 24.10% of the total population, respectively [15]. Education and health care remain limited due to the low economic power of the population. Maternal and child mortality rates are high, at 371 per 100,000 live births and 61 per 1,000 infants under five, respectively [16]. In the last 20 years, Burkina Faso has successively carried out three major health financing reforms to improve maternal and child health. These consisted of a national subsidy policy for maternity care consisting of an 80% reduction of fees at health centres, district hospitals, and referral hospital levels (2006 to 2015); a results-based financing scheme (2011 to 2016); and a free healthcare policy for maternal and child care introduced in all public health facilities and some confessional accredited facilities from April 2016. The most recent policy aims to facilitate access to healthcare by eliminating user fees at the point of care. In turn, it should increase the use of services by the target populations and improve health outcomes. This free healthcare policy is operational in all public health facilities (primary health centres, district, regional, and national hospitals) and some confessional accredited facilities for childhood diseases, pregnancy, delivery, and postpartum care, as well as screening and treatment of precancerous cervical lesions and breast examination. The policy provides for waiving all the direct in-facility costs, including hospitalization, medicine and consumables, and medical acts, i.e. consultations, postoperative care, lab exams, and referral transportation costs [17]. A standard operating procedure guarantees the management and outlines the method of its implementation. Since the policy’s launch, internal reports of the Ministry of Health reported an increase in the use of services. However, at the same time, various problems related to the implementation have been raised, including insufficient monitoring and evaluation and various irregularities in the care of patients in certain health facilities. In addition, launching the free healthcare policy for maternal and child health has coincided with the amplification of terrorist attacks in several health districts. This could further compromise access and availability of healthcare services to target populations, especially vulnerable and marginalized groups, even if the services are free. This contrasting picture reinforces the need to analyse, at the country level, the effects of the free healthcare policy on service use and health outcomes.

### Study design

This study used a variety of quantitative approaches and a desk review of strategic and programmatic documents to address the research objectives. The effects of the user fee removal policy were assessed based on a self-controlled interrupted time-series design to investigate changes in health service use and outcomes for mothers and children. Data on health service use and outcomes for up to 33 months after the user fee removal policy was compared to 99 monthly pre-policy introduction data in 43 of the country’s 70 health districts. The excluded districts were either newly created (11 health districts) or had incomplete data. In addition, an analysis of household out-of-pocket expenditures related to delivery was conducted to examine the effects of the policy reform on current household expenditures for delivery.

### Data sources

The data used to investigate the effects of the user fee removal on health service use, outcome indicators, and efficiency in providing healthcare services by districts were retrospectively collected from the National Health Information System (NHIS), for which data reliability has been acknowledged [18–20]. The data were compiled to form a reliable continuous time series from January 2008 to December 2018. They included monthly counts of facility-based and cesarean deliveries, second antenatal consultations, complicated deliveries, consultations for children under five, intrahospital infant mortality, and severe malaria in children under five. In addition, distance to facilities, appropriate population sizes (including children under five), women of childbearing age, the total population from the respective catchment areas, and the number of health personnel, and facilities per district were also gathered from NHIS.

The data used to measure the effectiveness of the policy in eliminating user fees were prospectively collected from a cross-sectional household survey. The dataset was constituted between October and November 2020. It comprised cost data for 797 users of the free healthcare services, including beneficiaries who resorted to facilities for delivery care, those who received infant care, and those who received various other health services covered by the policy (antenatal and postnatal care). All beneficiaries were systematically recruited, upon consenting, from 15 public health facilities (including two tertiary-level hospital facilities, two regional hospital facilities, and eight district-level facilities) from four out of the 13 health regions of the country. The health facilities were selected based upon a hierarchical classification of the country’s health facilities into four homogeneous groups based on their caesarean rate, the proportion of assisted deliveries, the average distance to a health facility, and the poverty rate.

All the data was collected by experienced interviewers using a pilot-tested quantitative instrument. The survey tool included questions about healthcare seeking, service utilisation, costs of seeking care, and healthcare costs (e.g., consultation, testing, medication, etc.). Data were available for analysis for 244 women with different delivery outcomes (cesareans, complicated and uncomplicated deliveries), 401 records on access to child care, and 152 records on access to other exempted care (antenatal and postnatal care).

### Study variables

Two categories of variables, including access and health indicators, were used to assess the impact of the user fee removal policy. Service use indicators included assisted deliveries, cesarean sections, second antenatal consultations, complicated deliveries, and consultations for children under five. Health outcome indicators included deaths from severe malaria in children under five, intrahospital infant deaths, and a cost evaluation of the current household expenditures on delivery.

### Statistical analysis

#### Model specification

Since indicators of interest were expressed as monthly counts, likelihood models were deemed appropriate to investigate the effects of user fee removal (UFR) on the use of healthcare services by women and children and the resulting outcomes compared to linear regressions. There were also no comparison districts because of the nationwide nature of the free healthcare policy, which prevented us from using quasi-experimental approaches to analyze its effects. Since successive healthcare policies had been implemented, interrupted time-series techniques appeared appropriate. We used a self-controlled case series design to compare the rate in a given indicator of interest after the user fee removal to its rate before the introduction of the policy (comparator period). Interrupted time-series analyses were performed using a dataset comprising 132 monthly observation points from January 2008 to December 2018. The conditional (fixed) Poisson regression (Stata Xtpoisson) was used to calculate incidence rate ratios with robust standard errors to compare the rate of a given indicator after/before the health policy reform by applying the following equation:

$$\begin{array}{l}{Y_{it}} = {\beta _0} + \beta _1^*time + \beta _2^*UF{R_{it}} + \beta _3^*postUF{R_{it}}\\+ \beta _4^*UF{R_{it}}*HW{d_{it}} + \beta _5^*UF{R_{it}}*P{d_{it}}\\+ + \beta _6^*Se{c_{it}} + Po{p_{it}} + {\varepsilon _t}\end{array}$$

Where $${Y}_{it }$$was the outcome variable in district $$i$$ during time $$t,$$ that is, the monthly count of an indicator chosen among the selected study indicators; time represented the monthly periods$$,$$ that is, a continuous variable indicating time from the start of the study up to the end of the observed period; $${UFR}_{it}$$ was a dichotomous variable denoting pre- and post-policy changes, with 0 equaling ‘no $$\text{U}\text{F}\text{R}$$’ at time $$t$$ in district $$i$$, and 1 for the presence of ‘$$\text{U}\text{F}\text{R}$$’ at time $$t$$ in district $$i$$. $${Pop}_{it}$$ was an offset representing the target population size at time $$t$$ in district $$i$$. The expected number of assisted deliveries was used as an offset in the analysis of assisted deliveries, cesarean sections, second antenatal consultations, and complicated deliveries. The total population of children under five was used as an offset in the analysis of under-five consultations and for under-five and intrahospital mortalities. $${HWd}_{it}$$, $${Pd}_{it}$$$${\text{a}\text{n}\text{d} Sec}_{it}$$were also dichotomous variables controlling for health personnel density, the average distance to health facilities and security level in a given health district, respectively. Section labelled **effects of covariates **further explains the coding of these three covariates.

${\beta }_{0}$ was the average baseline level in the given indicator at time 0 in the 43 health districts; ${\beta }_{1}$ estimated the structural trend or pre-policy slope independently from the policy reform being active or not; ${\beta }_{2}$ estimated the level of change in the outcome of interest after the adoption of the policy; ${\beta }_{3}$ reflected the change in trend in outcome after the adoption of the policy reform; and ${{\beta }}_{4},{{\beta }}_{5}$ and ${{\beta }}_{6}$ controlled the effect of health worker density, distance to the health facility, and security level, respectively. All estimations were adjusted for the calendar month (to control for seasonality). We reported effects (estimated *β*) as incidence rate ratios (IRR) with 95% confidence intervals to ease interpretation. An IRR value less than 1 meant a reduction (protective effect of the user fee removal policy of 1-IRR), while an IRR value greater than 1 meant an increase in the given indicator.

#### Effects of covariates

Contextual and health service variables are essential in explaining healthcare and its outcomes. For example, the empirical literature has reported that healthcare facilities with a higher density of human resources were more likely to show lower maternal, infant, and under-five mortalities [21]. Literature has also shown that populations closer to health facilities were more likely to use them than distant populations [22]. Because of this, we added two time-invariant covariates in our regression models. The first variable, which described accessibility to services, was defined as the fraction of the population living more than 10 km from each health centre. This variable was coded 1 for health facilities where more than half of the target population lived further than 10 km from the facility and 0 otherwise. The second variable related to health personnel density was defined as the number of healthcare personnel per 1,000 inhabitants from each health district catchment’s total population. The latter variable was further dichotomized to help disentangle the potential effect of health personnel density; a value of 1 denoted facilities with a higher density of health care personnel, and 0 otherwise. In line with previous research, a value of 0.45 denoted facilities with a higher density of healthcare personnel and 0 otherwise [12]. In addition, the precarious security context in some areas of the country due to terrorist attacks appeared to be a significant constraint in implementing national healthcare strategies. For this reason, a dummy covariate which captured the occurrence of terrorist attacks (i.e. monthly discrete events) in each district was added to the model. A value of 0 denoted districts with compromised security, that is, closed health facilities or operating at a minimum, and 1 otherwise.

### Effectiveness of cost removal

The effect of user fee removal on household costs for the delivery, care for children and other exempted services was analyzed by estimating the mean cost borne by households to access them. Only direct medical costs were considered in estimating total costs borne by households. These included consultations, expenses for medicines and consumables, laboratory tests, ultrasounds, hospitalization, and payments made outside the health facility, such as purchasing drugs that were out of stock in the health facility’s pharmaceutical store. Informal payments borne by households were also analyzed. The mean out-of-pocket cost households bear to access any exempted service was then estimated as the average cost in the sample using a two-part (logistic and OLS) modelling regression. This mean cost was further disaggregated into mean cost per delivery, care for children and other exempted care (combining antenatal and postnatal care). In theory, the cost of any exempted care should have been null under the free healthcare policy for the abovementioned services. To assess the effectiveness of the policy in removing household costs, we compared the actual payment of delivery to the theoretical nil costs if the policy was well implemented. We also analyzed the evolution of the delivery costs paid by households over time, adjusting mean and median costs borne by households before the free healthcare policy as given in Ganaba, Ilboudo [20] and comparing them with mean and median delivery costs under the free healthcare policy. We only did so for delivery care since delivery costs were well documented in the literature. To ease the comparison of cost data of different periods, we converted all costs before and after the introduction of the free healthcare policy into their equivalent values in US$ (2018), adjusting for inflation in US$, with US$1 = 559 FCFA [23]. All analyses were performed on Stata 13.

## Ethical consideration

The Ethics Committee for Health Research (Comité d’Éthique pour la Recherche en Santé) approved the study protocol and tools on its deliberation on September 2, 2020. In addition, all administrative authorizations were obtained before conducting the interviews. All the study participants provided written informed consent.

## Results

### Trends in selected indicators of service use

**Fig. 1:**
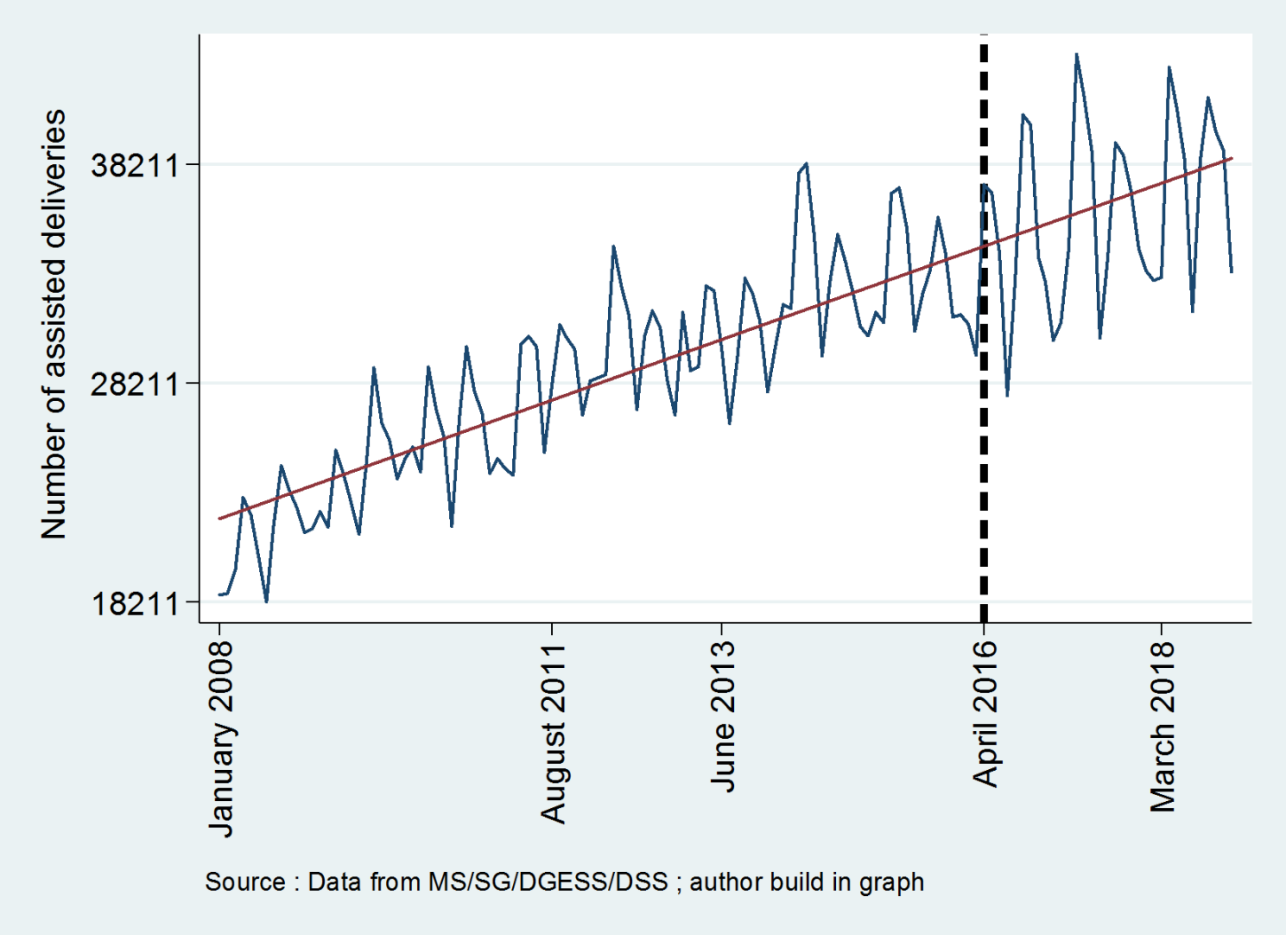
Trend in assisted deliveries

**Fig. 2:**
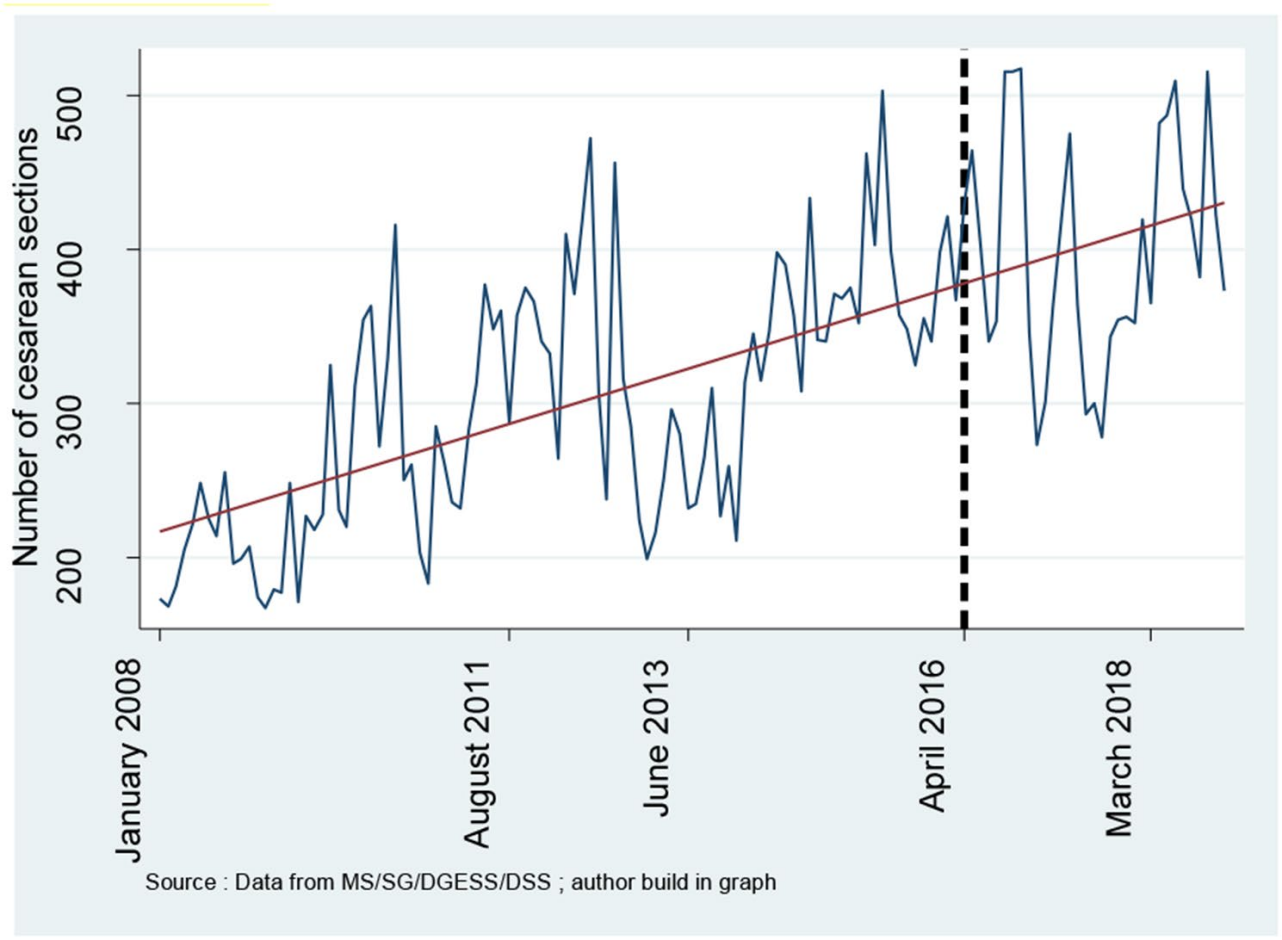
Trend in cesarean sections

**Fig. 3:**
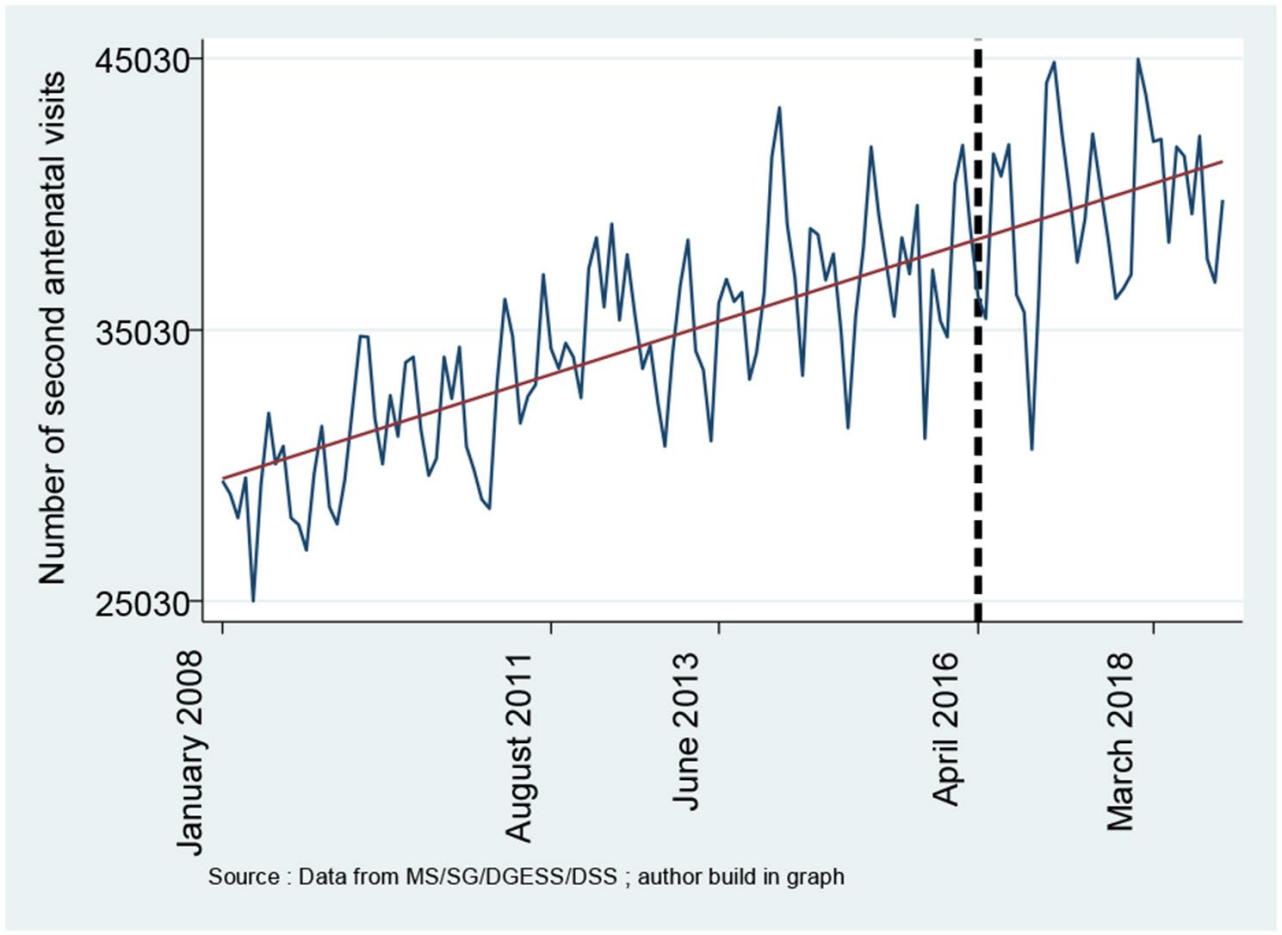
Trend in second antenatal visits

**Fig. 4:**
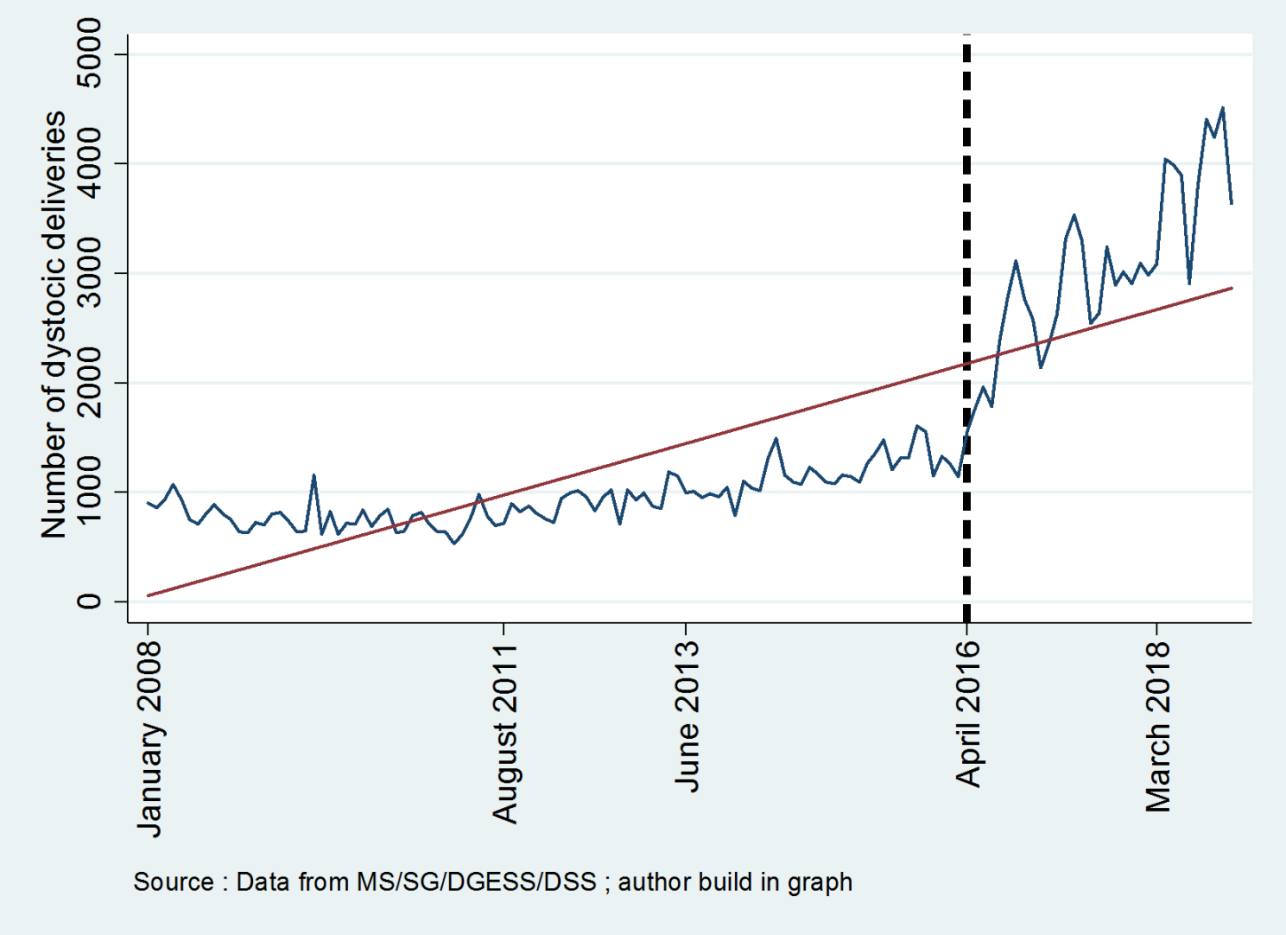
Trend in complicated deliveries

**Fig. 5:**
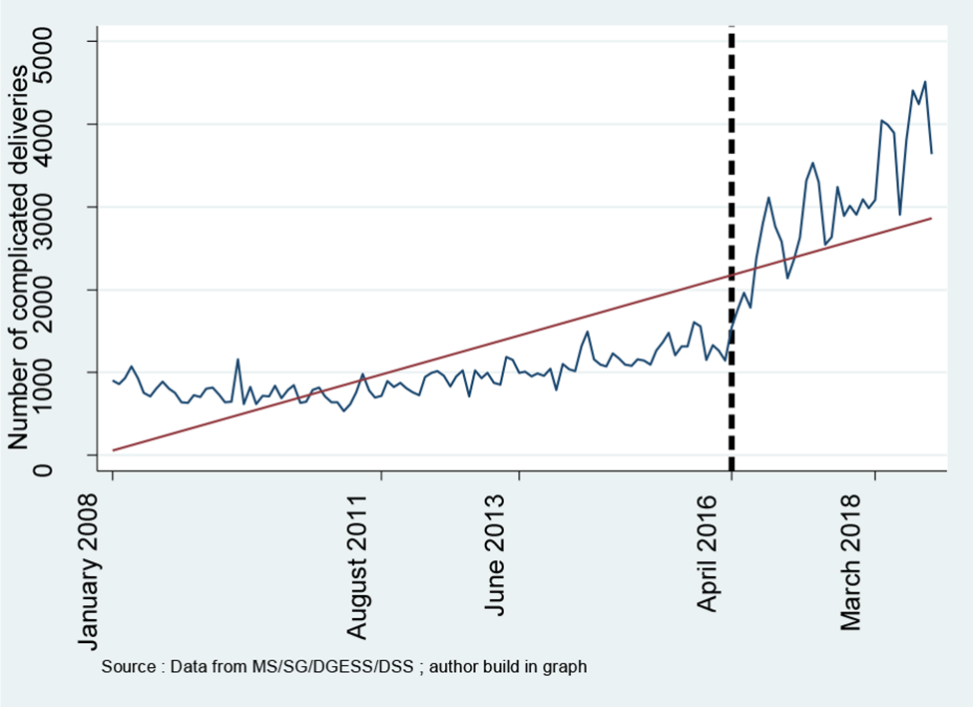
Trend in consultations for children less than five-years old

**Figures **1, 2, 3, 4 and 5 report the use of healthcare facilities for assisted deliveries, cesarean sections, second antenatal consultations, complicated deliveries, and consultations for children under five years old before and after the introduction of the free healthcare policy for maternal and children’s health. All five figures exhibit relative increases in health service utilization for these indicators.

### Trends in selected health outcomes

**Fig. 6:**
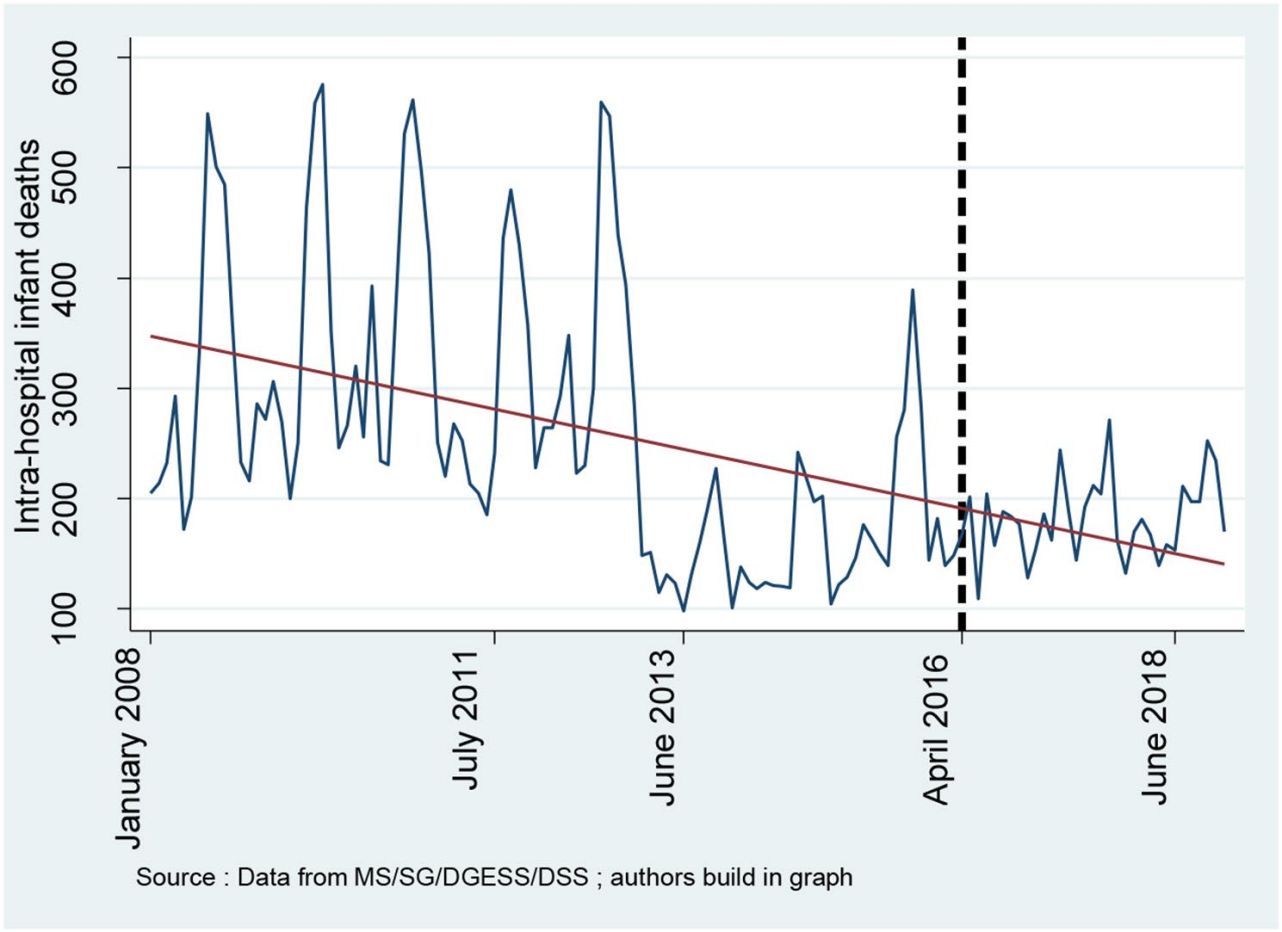
Trend in intrahospital infant deaths

**Fig. 7:**
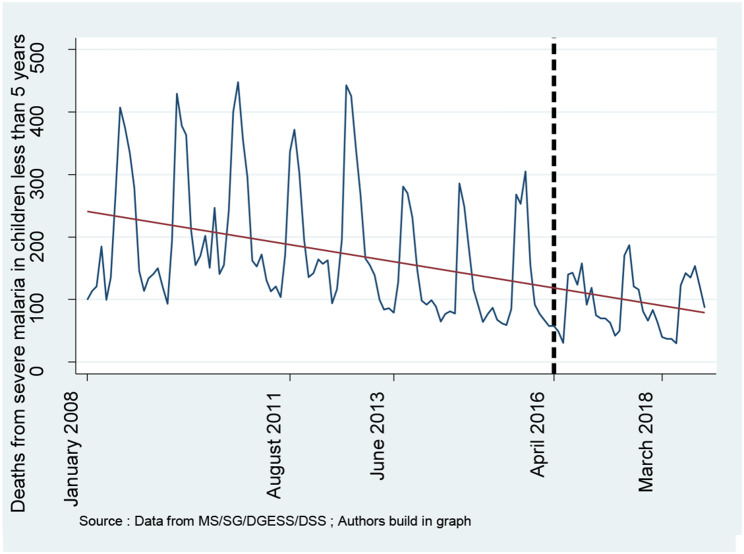
Trend in deaths from severe malaria in children under five years old

**Figures **6 and 7 show monthly intra-hospital mortality and mortality from severe malaria in children under five, respectively. Both figures show relative decreases in intrahospital mortality and mortality from severe malaria in children under five.

### Effects on the use of services

Table 1 reports the effects of the user fee removal policy on assisted deliveries, cesareans, second antenatal visits, complicated deliveries, and consultations for children under five years old. The user fee removal policy showed significant effects at a 95% confidence level only for consultations for children under five. The findings indicate that the health policy reform has increased the use of healthcare facilities for consultations for children under five years old by more than 800% (IRR: 9.66; 95% CI [1.517–61.56]). The policy did not significantly affect the use of healthcare services for assisted deliveries, cesareans, complicated deliveries, and second antenatal visits.

**Table 1:** Effects of user fee removal policy on selected indicators of the use of services

	Model 1			Model 2		
	IRR	95% CI		IRR	95% CI	
**Assisted deliveries**						
Constant${\beta }_{0}$	1.001***	(1.001–1.001)		1.001***	(1.001–1.001)	
Secular trend${\beta }_{1}$	1.003***	(1.003–1.003)		1.003***	(1.002–1.004)	
Change in level${\beta }_{2}$	1.126***	(1.084–1.169)		1.126	(0.615–2.062)	
Change in trend${\beta }_{3}$	0.996***	(0.996–0.996)		0.996	(0.991–1.001)	
Distance effect modification	1.245***	(1.230–1.260)		1.245	(0.911–1.702)	
Health personnel effect modification	1.068***	(1.049–1.087)		1.068	(0.909–1.255)	
Security effect modification	1.004	(0.999–1.009)		1.004	(1.001–1.001)	
**Cesarean sections**						
Constant${\beta }_{0}$	0.999***	(0.999-1.000)		0.999**	(0.999–0.999)	
Secular trend${\beta }_{1}$	1.008***	(1.007–1.009)		1.008***	(1.002–1.013)	
Change in level${\beta }_{2}$	0.889	(0.635–1.245)		0.889	(0.085–9.310)	
Change in trend${\beta }_{3}$	1.001	(0.997–1.004)		1.001	(0.979–1.022)	
Distance effect modification	1.120**	(1.007–1.246)		1.120	(0.504–2.490)	
Health personnel effect modification	0.320***	(0.278–0.369)		0.320***	(0.156–0.651)	
Security effect modification	1.414***	(1.344–1.490)		1.415*	(0.975–2.054)	
**Second antenatal consultations**						
Constant${\beta }_{0}$	1.001***	(1.001–1.001)		1.001***	(1.001–1.001)	
Secular trend${\beta }_{1}$	1.002***	(1.002–1.002)		1.002**	(1.000-1.003)	
Change in level${\beta }_{2}$	1.357***	(1.309–1.406)		1.357	(0.788–2.337)	
Change in trend${\beta }_{3}$	0.995***	(0.995–0.996)		0.995*	(0.990–1.001)	
Distance effect modification	0.995	(0.984–1.006)		0.995	(0.875–1.132)	
Health personnel effect modification	1.016*	(0.999–1.033)		1.016	(0.875–1.179)	
Security effect modification	0.894	(0.890–0889)		0.894*	(0.795–1.006)	
**Complicated deliveries**						
Constant${\beta }_{0}$	1.000***	(1.000–1.000)		1.000**	(1.000–1.000)	
Secular trend${\beta }_{1}$	1.012***	(1.011–1.012)		1.008**	(1.003–1.014)	
Change in level${\beta }_{2}$	1.406***	(1.177–1.679)		1.406	(0.266–7.422)	
Change in level${\beta }_{3}$	1.002*	(1.000-1.003)		1.002	(0.985–1.018)	
Distance effect modification	0.903***	(0.863–0.965)		0.903	(0.712–1.146)	
Health personnel effect modification	0.264***	(0.249–0.281)		0.264**	(0.110–0.634)	
Security effect modification	1.171***	(1.140–1.204)		1.171	(0.630–1.654)	
**Consultations for children under five**						
Constant${\beta }_{0}$	1.000***	(1.000–1.000)		1.000	(0.999-1.000)	
Secular trend${\beta }_{1}$	1.008***	(1.007–1.008)		1.008*	(1.003–1.014)	
Change in level${\beta }_{2}$	9.663***	(7.549–12.37)		9.66**	(1.517–61.56)	
Change in trend${\beta }_{3}$	0.897***	(0.984–0.989)		0.986	(0.970–1.004)	
Distance effect modification	0.420***	(0.389–0.453)		0.419	(0.152–0.160)	
Health personnel effect modification	0.548***	(0.517–0.580)		0.548	(0.387–0.775)	
Security effect modification	2.056***	(1.930–2.195)		2.059	(0.881–4.808)	

Model 1 = before correction of heteroscedasticity and autocorrelation

Model 2 = after correction of heteroscedasticity and autocorrelation

IRR = Incidence rate ratio

Significance level: *** p < 0.01; ** p < 0.05; * p < 0.10

### Effects on health outcomes

Table 2 shows the effects of the user fee removal policy on intra-hospital infant mortality and mortality from severe malaria in children under five. The findings show that the free healthcare policy has significantly decreased mortality from severe malaria in children under five by 92.60% (IRR: 0.074; 95% CI [0.011–0.472]). Moreover, the findings indicate that the user fee removal policy has also reduced intrahospital infant mortality by 68.60% (IRR: 0.314; 95% CI [0.044–2.211]). However, the result was not significant at the 5% confidence level.

**Table 2:** Effects of user fee removal policy on selected health outcomes

	Model 1			Model 2	
	IRR	95% CI		IRR	95% CI
**Intra-hospital infant deaths**					
Constant${\beta }_{0}$	6.600***	(5.484–7.942)		6.600***	(2.983–14.60)
Secular trend${\beta }_{1}$	0.990***	(0.989–0.991)		0.990***	(0.986–0.994)
Change in level${\beta }_{2}$	0.314***	(0.203–0.486)		0.314	(0.044–2.211)
Change in trend${\beta }_{3}$	1.018***	(1.014–1.023)		1.018**	(1.000-1.037)
Distance effect modification	0.644***	(0.567–0.731)		0.644	(0.347–1.195)
Health personnel effect modification	0.711***	(0.600-0.843)		0.711	(0.426–1.186)
Security effect modification	1.090**	(1.026–1.158)		1.090	(0.753–1.577)
**Mortality from severe malaria in children under five years**					
Constant${\beta }_{0}$	1.000***	(1.000–1.000)		1.000***	(1.000–1.000)
Secular trend${\beta }_{1}$	0.991***	(0.990–0.992)		0.991***	(0.987–0.994)
Change in level${\beta }_{2}$	0.074***	(0.041–0.133)		0.074**	(0.011–0.472)
Change in trend${\beta }_{3}$	1.032***	(1.027–1.038)		1.032***	(1.013–1.052)
Distance effect modification	0.706***	(0.595–0.837)		0.706	(0.456–1.092)
Health personnel effect modification	0.461***	(0.376–0.563)		0.462**	(0.239–0.889)
Security effect modification	0.934*	(0.865–1.008)		0.934	(0.663–1.315)

Model 1 = before correction of heteroscedasticity and autocorrelation

Model 2 = after correction of heteroscedasticity and autocorrelation

IRR = Incidence rate ratio

Significance level: *** p < 0.01; ** p < 0.05; * p < 0.10

### Effects of covariates

The findings show that the effects of the intervention on cesareans and mortality from severe malaria in children under five years old were 68.00% and 53.80%, significantly lower in districts with higher workforce density (IRR: 0.320; 95% CI (0.156–0.651) and IRR: 0.462; 95% CI (0.239–0.889), respectively). The effects of the intervention on complicated deliveries and intrahospital infant mortality were also lower in districts with a higher workforce. Concerning the other studied indicators, the effects of the user fee removal seem higher in districts with a higher workforce. However, these results were not statistically significant at the 95% confidence level. The findings also show that the effects of the user fee removal on assisted deliveries and cesareans were higher in districts with highly dispersed populations. The effects of user fee removal on second antenatal consultations, complicated deliveries, intrahospital mortality, and mortality from severe malaria in children under five were lower in districts with highly dispersed populations. However, the results were insignificant. Finally, except for second antenatal consultations and mortality from severe malaria in children under five, the effects of the user fee removal policy on all other studied indicators seemed higher in districts with non-compromised security.

### Effects on household costs

Table 3 reports the average cost borne by households for institutional deliveries, care for children, and other exempted healthcare services. Independent of the covered service, the mean cost to households under the current free healthcare policy was US$11.76. Disaggregated figures showed that the mean costs to households were US$21.24, US$5.76 and US$13.33 for delivery, care for children, and other exempted services, respectively. The results show a significant reduction in household costs for the delivery, from US$70.90 (before the EmOC subsidy policy, i.e., user fee charged at the time) to US$21.24 under the free healthcare policy for maternal and child health. The mean household costs for delivery were US$21.78 and US$28.33 in the district and regional hospital facilities before the free healthcare policy and US$21.24 under the policy. Interestingly, the findings also show null median costs for delivery care, care for children and other exempted services, including antenatal care and postnatal.

**Table 3:** Mean costs borne by households for accessing in 2018 US$

	Mean cost^b^	SD	Median
**Before the free healthcare policy**			
Before EmOC subsidy policy^a,b^	70.90	NR	NR
During EmOC subsidy policy^a,b^			
Primary health center	2.85	NR	1.90
District hospital	21.78	NR	17.41
Regional hospital	28.33	NR	23.55
**Free healthcare policy** ^**c**^	11.76	0.71	0.00
Delivery care	21.24	1.74	0.00
Care for children	5.76	1.08	0.00
Other exempted care	13.33	1.60	0.00

^a^Mean delivery cost primary cost data taken from Ganaba et al. (2016) ; ^b^Mean cost borne by households; ^b^Mean cost borne by households irrespective of the level of the facility ; NR = Not reported in original papers; SD = Standard deviation

## Discussion

This study is the first attempt to investigate at a national level the effects of the free healthcare policy for maternal care and care of children under five years old in Burkina Faso. This work is unique since it comprehensively studied the policy’s effects on nationwide service use and health outcomes. Most previous studies have limited scope and primarily assess free healthcare experiments [12, 19, 24]. In addition, this study critically interrogated the effects of the free healthcare policy for maternal and child health in light of the contextual situation of the country, integrating the effects of insecurity. None of the previous studies has attempted to take the effects of insecurity into account. Because of its scope, the present investigation brought several insights worthy of discussion.

### Effects on the use of services

The findings show a significant increase in the use of healthcare facilities for consultations for children under five years old. This finding is consistent with previous research that found increased use of healthcare services by children following the introduction of the free healthcare policy for maternal and child health in Burkina [12, 25, 26]. The finding also aligns with numerous review papers demonstrating increased service use following user fee removal [27–29]. Though not significant at the 95% confidence level, the findings also show increased use of healthcare facilities for assisted deliveries, second antennal visits, complicated deliveries, and reduced cesarean sections. The increased use of health facilities for antenatal visits and assisted deliveries was consistent with findings from numerous previous studies. Research has shown that user fee removal is associated with increased utilization of health facilities for delivery in Sudan [30] and some pilot experiments in Burkina Faso [19, 24]. The apparent increase in antenatal consultations and assisted deliveries seems also to align with findings from Hangoma, Robberstad [31], Xu, Evans [32], and John [30]. Unlike a previous review finding that the abolition of user fees led to increased cesarean sections [33], this study showed a reduction in cesarean sections. This divergent result could be explained by the increased utilization of health facilities for antenatal consultations and assisted deliveries. Antenatal consultations are critical for the early detection of abnormalities and signs of complications as well as proper management of the delivery. Increased antenatal consultations and assisted deliveries could be why more complicated deliveries were managed in hospital facilities. These, in turn, may well explain the reduction in cesareans that would have otherwise been performed to save lives.

### Effects on health outcomes

The introduction of the policy significantly reduced mortality from severe malaria in children under the age of five. Though insignificant, the results also indicated a decrease in intrahospital infant mortality. The increased use of consultative care for children under five could mediate the reduced mortality from severe malaria in children under five. A substantial body of research has demonstrated the association between access to healthcare and reduced mortality in children under five years old [22, 34, 35]. In Burkina Faso, many researchers that have evaluated the impacts of the introduction of the free healthcare policy for maternal and child health reported increased use of healthcare services by children in rural [25] and in both rural and urban areas [12, 26]. Our findings align with a previous experiment that showed increased use of consultative care services for children under five years old in Kaya, Burkina Faso [18]. Increased use of health facilities for curative and preventive care after removing user fees has also been reported by several studies in many other places [27, 29, 32, 36], thereby indicating a significant contribution of the policy to the improvement of children’s health. Evidence of increased use of health services by children may well contribute to reducing deaths from severe malaria through promptly initiating appropriate care. Numerous other studies have shown that delivery in a health facility with a skilled provider reduces early neonatal and infant mortality [37–39]. This study also showed increased institutional deliveries, though not significantly. Several other studies conducted in Burkina Faso have demonstrated increased facility-based deliveries following the launching of the free healthcare policy for maternal and child health [24]. Increased hospital deliveries may have also contributed to reducing the overall child mortality, including mortality caused by severe malaria in children under five.

### Effects on household costs

The findings show that the user fee removal policy has further reduced costs borne by households compared to cost levels before the introduction of the free health care policy. It has even eliminated delivery costs and care costs for children and other exempted healthcare services for half of the households. Previous studies conducted in Burkina Faso also demonstrated that the fee exemption policy, whether partial or not, was ineffective in eliminating costs as it was supposed to [20, 40]. The user fee removal policy has only removed costs for half of the studied population, as shown by the median cost of 0. However, it still needs to remove all the direct costs related to delivery, child care and other exempted services for all households. This finding is remarkably consistent with a study in Burkina Faso which found that the user fee removal policy did not remove all the direct costs it was supposed to [40]. The finding is also in line with previous other research studies, which have found that the introduction of user fee removal policies has failed to remove household costs for care in Uganda [32], Ghana [41] and more recently in Zambia [31].

## Limitations

This study had several restraints. Though there was a gentle application of time-series techniques in this investigation, the absence of a control group may have weakened the power of the findings. Secondly, the study focused on measuring the effects of the user fee removal policy on the use of services and resulting outcomes using NHIS data. Though the country’s NHIS has often been acknowledged as appropriate [12, 24], it does not collect users’ socio-economic characteristics. Because of this, it was not possible to conduct a detailed analysis of the distributive impact of user fee removal as described by a previous study [42]. Third, several supply and demand side factors affecting health service use were controlled for, including health personnel density, distance, and insecurity. However, the likelihood that some critical factors affecting the use of health services, such as cultural factors or quality of services, may have yet to be accounted for since NHIS does not collect this data routinely. These also may have affected the study’s findings.

## Conclusions

This paper contributes to the body of evidence on the cost, and effects of user fee removal policies on maternal and children’s health. The originality of this study lies in the fact that it evaluated the effect of the health policy reform at the country level, taking into account contextual factors such as insecurity. Insecurity has never been incorporated in previous evaluations.

The results suggest that user fee removal policies for maternal and children’s health increased consultations for children under five and reduced mortality from severe malaria in children under five. The findings also show that the policy eliminated healthcare costs for half of the people. There seem to be indications of increased service use for the other studied indicators. Given the positive effects, the findings of this investigation support the pursuit of its implementation.

## Electronic supplementary material

Below is the link to the electronic supplementary material.

Supplementary Material 1

## Data Availability

The datasets used and/or analyzed during the current study are available from the corresponding author on reasonable request.

## Abbreviations

BCEAO: West African Economic and Monetary Union Central Bank
CI: Confidence Interval
EMOC: Emergency Obstetric Care
FCFA: Franc de la Communauté Financière de l’Afrique
GDP: Gross Domestic Product
GNI: Gross National Income
IMF: International Monetary Fund
IRR: Incidence Rate Ratio
MoH: Ministry of Health
NHIS: National Health Information System
OLS: Ordinary Least Square
PNDES: National Plan for Economic and Social Development
UFR: User Fee Removal
US$: United States Dollar
