# Supplementary material for: Effects of the free healthcare policy on maternal and child health in Burkina Faso: a nationwide evaluation using interrupted time-series analysis
Source: Health Econ Rev. 2023 May 5;13:27. doi: 10.1186/s13561-023-00443-w (PMC10161454; doi:10.1186/s13561-023-00443-w)
Supplement: Supplementary file 1 — Supplementary Material 1 [file 13561_2023_443_MOESM1_ESM.docx]

**Effects of the free healthcare policy on maternal and child health in Burkina Faso: a nationwide evaluation using interrupted time-series analysis**

**Abstract**

**Background**

Burkina Faso has recently instituted a free healthcare policy for women and children under five. This comprehensive study examined the effects of this policy on the use of services, health outcomes, and removal of costs.

**Methods**

Interrupted time-series regressions were used to investigate the effects of the policy on the use of health services and health outcomes. In addition, an analysis of household expenditures was conducted to assess the effects of spending on delivery, care for children, and other exempted (antenatal, postnatal, etc.) services on household expenditures.

**Results**

The findings show that the user fee removal policy significantly increased the use of healthcare facilities for child consultations and reduced mortality from severe malaria in children under the age of five years. It also has increased the use of health facilities for assisted deliveries, complicated deliveries, and second antenatal visits, and reduced caesarian deliveries and intrahospital infant mortality, although not significantly. While the policy has failed to remove all costs, it decreased household costs to some extent. In addition, the effects of the user fee removal policy seemed higher in districts with non-compromised security for most of the studied indicators.

**Conclusions**

Given the positive effects, the findings of this investigation support the pursuit of implementing the free healthcare policy for maternal and child care.

**Keywords**: User fee removal policy, effects, costs, segmented regression, Burkina Faso

1. **Introduction**

Over the last two decades, the improvement of maternal health and the health of children under five has been a critical priority for the international community through Millennium Development Goals 4 and 5([1](#_ENREF_1)). Despite this, the burden of maternal and child mortality has remained extremely high in many countries, particularly in sub-Saharan Africa. Several barriers, including delayed access to emergency obstetric care, especially caesarian sections, and widespread poverty, are significant obstacles to improving health outcomes for mothers and children. In settings with pervasive poverty, user charges have been an impediment for many households, preventing women from seeking qualified care during pregnancy or delivery, even in the event of complications ([2](#_ENREF_2)). Those who access care experience substantial difficulties paying for hospital fees and often resort to coping strategies such as selling assets, borrowing from friends or family members or accruing new debts to meet the costs ([3](#_ENREF_3)). These may lead to long-term negative consequences ([4](#_ENREF_4), [5](#_ENREF_5)).

Several sub-Saharan countries, including Burkina Faso, Burundi, Gabon, Ghana, Kenya, Liberia, Nigeria, Senegal and Uganda, have substantially or entirely removed user fees for maternal care and children under five ([6-9](#_ENREF_6)). The rationale behind such policies is the broad recognition that user fees constitute a significant financial barrier to accessing healthcare, especially for vulnerable groups such as women, children and the poorest segment of the population ([8](#_ENREF_8), [9](#_ENREF_9)). User fee removal policies aim to facilitate access to care by lowering/alleviating financial barriers at the point of care. Removal of the user fees, in turn, will contribute to increased use of qualified services and, ultimately, to improved health outcomes ([10](#_ENREF_10)).

Impacts of user fee removals still need to be well documented in Burkina Faso. Before the introduction of the free healthcare policy for maternal and child health in Burkina Faso, only a few studies, using interrupted time series, evaluated isolated experiments to reduce or eliminate the cost of child healthcare based on limited temporal and/or geographical coverage in the North or Sahel regions ([11](#_ENREF_11), [12](#_ENREF_12)). A more recent study has examined the cross effects of performance-based financing and user fee removal policies in selected districts in Burkina Faso ([13](#_ENREF_13)). They showed that removing user fees had increased the use of health services for consultations in children under five. None of these previous studies has, at the national level, thoroughly investigated the effects of the free healthcare policy on the use of healthcare services and outcomes. In addition, they have yet to investigate the effectiveness of the policy in eliminating costs. This study investigates the effects of user fee removal on health services usage and outcomes at the country level. It also analyzes the effectiveness of the policy in removing household costs.

1. **Methods**

**2.1 Study setting and health policy initiatives**

Burkina Faso is a low-income landlocked country in West Africa with a total population of 20,505,155 as of 2021 ([14](#_ENREF_14)). Women of reproductive age and children constitute 17.43% and 24.10% of the total population, respectively ([15](#_ENREF_15)). Education and health care remain limited due to the low economic power of the population. Maternal and child mortality rates are high, at 371 per 100,000 live births and 61 per 1,000 infants under five, respectively ([16](#_ENREF_16)). In the last 20 years, Burkina Faso has successively carried out three major health financing reforms to improve maternal and child health. These consisted of a national subsidy policy for maternity care consisting of an 80% reduction of fees at health centres, district hospitals, and referral hospital levels (2006 to 2015); a results-based financing scheme (2011 to 2016); and a free healthcare policy for maternal and child care introduced in all public health facilities and some confessional accredited facilities from April 2016. The most recent policy aims to facilitate access to healthcare by eliminating user fees at the point of care. In turn, it should increase the use of services by the target populations and improve health outcomes. This free healthcare policy is operational in all public health facilities (primary health centres, district, regional, and national hospitals) and some confessional accredited facilities for childhood diseases, pregnancy, delivery, and postpartum care, as well as screening and treatment of precancerous cervical lesions and breast examination. The policy provides for waiving all the direct in-facility costs, including hospitalization, medicine and consumables, and medical acts, i.e. consultations, postoperative care, lab exams, and referral transportation costs ([17](#_ENREF_17)). A standard operating procedure guarantees the management and outlines the method of its implementation. Since the policy's launch, internal reports of the Ministry of Health reported an increase in the use of services. However, at the same time, various problems related to the implementation have been raised, including insufficient monitoring and evaluation and various irregularities in the care of patients in certain health facilities. In addition, launching the free healthcare policy for maternal and child health has coincided with the amplification of terrorist attacks in several health districts. This could further compromise access and availability of healthcare services to target populations, especially vulnerable and marginalized groups, even if the services are free. This contrasting picture reinforces the need to analyse, at the country level, the effects of the free healthcare policy on service use and health outcomes.

**2.2 Study design**

This study used a variety of quantitative approaches and a desk review of strategic and programmatic documents to address the research objectives. The effects of the user fee removal policy were assessed based on a self-controlled interrupted time-series design to investigate changes in health service use and outcomes for mothers and children. Data on health service use and outcomes for up to 33 months after the user fee removal policy was compared to 99 monthly pre-policy introduction data in 43 of the country’s 70 health districts. The excluded districts were either newly created (11 health districts) or had incomplete data. In addition, an analysis of household out-of-pocket expenditures related to delivery was conducted to examine the effects of the policy reform on current household expenditures for delivery.

**2.3 Data sources**

The data used to investigate the effects of the user fee removal on health service use, outcome indicators, and efficiency in providing healthcare services by districts were retrospectively collected from the National Health Information System (NHIS), for which data reliability has been acknowledged ([18-20](#_ENREF_18)). The data were compiled to form a reliable continuous time series from January 2008 to December 2018. They included monthly counts of facility-based and caesarian deliveries, second antenatal consultations, complicated deliveries, consultations for children under five, intrahospital infant mortality, and severe malaria in children under five. In addition, distance to facilities, appropriate population sizes (including children under five), women of childbearing age, the total population from the respective catchment areas, and the number of health personnel, and facilities per district were also gathered from NHIS.

The data used to measure the effectiveness of the policy in eliminating user fees were prospectively collected from a cross-sectional household survey. The dataset was constituted between October and November 2020. It comprised cost data for 797 users of the free healthcare services, including beneficiaries who resorted to facilities for delivery care, those who received infant care, and those who received various other health services covered by the policy (antenatal and postnatal care). All beneficiaries were systematically recruited, upon consenting, from 15 public health facilities (including two tertiary-level hospital facilities, two regional hospital facilities, and eight district-level facilities) from four out of the 13 health regions of the country. The health facilities were selected based upon a hierarchical classification of the country’s health facilities into four homogeneous groups based on their caesarean rate, the proportion of assisted deliveries, the average distance to a health facility, and the poverty rate.

All the data was collected by experienced interviewers using a pilot-tested quantitative instrument. The survey tool included questions about healthcare seeking, service utilisation, costs of seeking care, and healthcare costs (e.g., consultation, testing, medication, etc.). Data were available for analysis for 244 women with different delivery outcomes (cesareans, complicated and uncomplicated deliveries), 401 records on access to child care, and 152 records on access to other exempted care (antenatal and postnatal care).

**2.4 Study variables**

Two categories of variables, including access and health indicators, were used to assess the impact of the user fee removal policy. Service use indicators included assisted deliveries, caesarian sections, second antenatal consultations, complicated deliveries, and consultations for children under five. Health outcome indicators included deaths from severe malaria in children under five, intrahospital infant deaths, and a cost evaluation of the current household expenditures on delivery.

**2.5 Statistical analysis**

**2.5.1 Model specification**

Since indicators of interest were expressed as monthly counts, likelihood models were deemed appropriate to investigate the effects of user fee removal (UFR) on the use of healthcare services by women and children and the resulting outcomes compared to linear regressions. There were also no comparison districts because of the nationwide nature of the free healthcare policy, which prevented us from using quasi-experimental approaches to analyze its effects. Since successive healthcare policies had been implemented, interrupted time-series techniques appeared appropriate. We used a self-controlled case series design to compare the rate in a given indicator of interest after the user fee removal to its rate before the introduction of the policy (comparator period). Interrupted time-series analyses were performed using a dataset comprising 132 monthly observation points from January 2008 to December 2018. The conditional (fixed) Poisson regression (Stata Xtpoisson) was used to calculate incidence rate ratios with robust standard errors to compare the rate of a given indicator after/before the health policy reform by applying the following equation:

$Y_{it}= \beta_{0}+ \beta_{1}^{*}time+ \beta_{2}^{*}{UFR}_{it}+ \beta_{3}^{*}{postUFR}_{it}+ \beta_{4}^{*}{UFR}_{it}*{HWd}_{it}+\beta_{5}^{*}{UFR}_{it}*{Pd}_{it}+{+ \beta_{6}^{*}{Sec}_{it}+Pop}_{it}+ \varepsilon_{t}$

Where $Y_{it}$was the outcome variable in district $i$ during time $t,$ that is, the monthly count of an indicator chosen among the selected study indicators; time represented the monthly periods$,$ that is, a continuous variable indicating time from the start of the study up to the end of the observed period; ${UFR}_{it}$ was a dichotomous variable denoting pre- and post-policy changes, with 0 equaling ‘no $\mathrm{UFR}$’ at time $t$ in district $i$, and 1 for the presence of ‘$\mathrm{UFR}$’ at time $t$ in district $i$. ${Pop}_{it}$ was an offset representing the target population size at time $t$ in district $i$. The expected number of assisted deliveries was used as an offset in the analysis of assisted deliveries, caesarian sections, second antenatal consultations, and complicated deliveries. The total population of children under five was used as an offset in the analysis of under-five consultations and for under-five and intrahospital mortalities. ${HWd}_{it}$, ${Pd}_{it}$ ${\mathrm{and}Sec}_{it}$were also dichotomous variables controlling for health personnel density, the average distance to health facilities and security level in a given health district, respectively. **Section 2.5.2** further explains the coding of these three covariates.

$\beta_{0}$ was the average baseline level in the given indicator at time 0 in the 43 health districts; $\beta_{1}$ estimated the structural trend or pre-policy slope independently from the policy reform being active or not; $\beta_{2}$ estimated the level of change in the outcome of interest after the adoption of the policy; $\beta_{3}$ reflected the change in trend in outcome after the adoption of the policy reform; and$\beta_{4},\beta_{5}$ and $\beta_{6}$ controlled the effect of health worker density, distance to the health facility, and security level, respectively. All estimations were adjusted for the calendar month (to control for seasonality). We reported effects (estimated *β*) as incidence rate ratios (IRR) with 95% confidence intervals to ease interpretation. An IRR value less than 1 meant a reduction (protective effect of the user fee removal policy of 1-IRR), while an IRR value greater than 1 meant an increase in the given indicator.

**2.5.2 Effect of covariates**

Contextual and health service variables are essential in explaining healthcare and its outcomes. For example, the empirical literature has reported that healthcare facilities with a higher density of human resources were more likely to show lower maternal, infant, and under-five mortalities ([21](#_ENREF_21)). Literature has also shown that populations closer to health facilities were more likely to use them than distant populations ([22](#_ENREF_22)). Because of this, we added two time-invariant covariates in our regression models. The first variable, which described accessibility to services, was defined as the fraction of the population living more than 10 km from each health centre. This variable was coded 1 for health facilities where more than half of the target population lived further than 10 km from the facility and 0 otherwise. The second variable related to health personnel density was defined as the number of healthcare personnel per 1,000 inhabitants from each health district catchment’s total population. The latter variable was further dichotomized to help disentangle the potential effect of health personnel density; a value of 1 denoted facilities with a higher density of health care personnel, and 0 otherwise. In line with previous research, a value of 0.45 denoted facilities with a higher density of healthcare personnel and 0 otherwise ([12](#_ENREF_12)). In addition, the precarious security context in some areas of the country due to terrorist attacks appeared to be a significant constraint in implementing national healthcare strategies. For this reason, a dummy covariate which captured the occurrence of terrorist attacks (i.e. monthly discrete events) in each district was added to the model. A value of 0 denoted districts with compromised security, that is, closed health facilities or operating at a minimum, and 1 otherwise.

**2.6 Effectiveness of cost removal**

The effect of user fee removal on household costs for the delivery, care for children and other exempted services was analyzed by estimating the mean cost borne by households to access them. Only direct medical costs were considered in estimating total costs borne by households. These included consultations, expenses for medicines and consumables, laboratory tests, ultrasounds, hospitalization, and payments made outside the health facility, such as purchasing drugs that were out of stock in the health facility’s pharmaceutical store. Informal payments borne by households were also analyzed. The mean out-of-pocket cost households bear to access any exempted service was then estimated as the average cost in the sample using a two-part (logistic and OLS) modelling regression. This mean cost was further disaggregated into mean cost per delivery, care for children and other exempted care (combining antenatal and postnatal care). In theory, the cost of any exempted care should have been null under the free healthcare policy for the abovementioned services. To assess the effectiveness of the policy in removing household costs, we compared the actual payment of delivery to the theoretical nil costs if the policy was well implemented. We also analyzed the evolution of the delivery costs paid by households over time, adjusting mean and median costs borne by households before the free healthcare policy as given in [Ganaba, Ilboudo (20)](#_ENREF_20) and comparing them with mean and median delivery costs under the free healthcare policy. We only did so for delivery care since delivery costs were well documented in the literature. To ease the comparison of cost data of different periods, we converted all costs before and after the introduction of the free healthcare policy into their equivalent values in US$ (2018), adjusting for inflation in US$, with US$1=559 FCFA ([23](#_ENREF_23)). All analyses were performed on Stata 13.

1. **Ethical consideration**

The Ethics Committee for Health Research (Comité d’Éthique pour la Recherche en Santé) approved the study protocol and tools on its deliberation on September 2, 2020. In addition, all administrative authorizations were obtained before conducting the interviews. All the study participants provided written informed consent.

1. **Results**

**4.1 Trends in selected indicators of service use**

**Figures 1** to **5** report the use of healthcare facilities for assisted deliveries, caesarian sections, second antenatal consultations, complicated deliveries, and consultations for children under five years old before and after the introduction of the free healthcare policy for maternal and children’s health. All five figures exhibit relative increases in health service utilization for these indicators.

**4.2 Trends in selected health outcomes**

**Figures 6** and **7** show monthly intra-hospital mortality and mortality from severe malaria in children under five, respectively. Both figures show relative decreases in intrahospital mortality and mortality from severe malaria in children under five.

**4.3 Effects on the use of services**

**Table 1** reports the effects of the user fee removal policy on assisted deliveries, cesareans, second antenatal visits, complicated deliveries, and consultations for children under five years old. The user fee removal policy showed significant effects at a 95% confidence level only for consultations for children under five . The findings indicate that the health policy reform has increased the use of healthcare facilities for consultations for children under five years old by more than 800% (IRR: 9.66; 95% CI [1.517-61.56]). The policy did not significantly affect the use of healthcare services for assisted deliveries, cesareans, complicated deliveries, and second antenatal visits.

**4.4 Effects on health outcomes**

**Table 2** shows the effects of the user fee removal policy on intra-hospital infant mortality and mortality from severe malaria in children under five. The findings show that the free healthcare policy has significantly decreased mortality from severe malaria in children under five by 92.60% (IRR: 0.074; 95% CI [0.011-0.472]). Moreover, the findings indicate that the user fee removal policy has also reduced intrahospital infant mortality by 68.60% (IRR: 0.314; 95% CI [0.044-2.211]). However, the result was not significant at the 5% confidence level.

**4.5 Effect of covariates**

The findings show that the effects of the intervention on caesarians and mortality from severe malaria in children under five years old were 68.00% and 53.80%, significantly lower in districts with higher workforce density (IRR: 0.320; 95% CI (0.156-0.651) and IRR: 0.462; 95% CI (0.239-0.889), respectively). The effects of the intervention on complicated deliveries and intrahospital infant mortality were also lower in districts with a higher workforce. Concerning the other studied indicators, the effects of the user fee removal seem higher in districts with a higher workforce. However, these results were not statistically significant at the 95% confidence level. The findings also show that the effects of the user fee removal on assisted deliveries and caesarians were higher in districts with highly dispersed populations. The effects of user fee removal on second antenatal consultations, complicated deliveries, intrahospital mortality, and mortality from severe malaria in children under five were lower in districts with highly dispersed populations. However, the results were insignificant. Finally, except for second antenatal consultations and mortality from severe malaria in children under five, the effects of the user fee removal policy on all other studied indicators seemed higher in districts with non-compromised security.

**4.6 Effect on household costs**

**Table 3** reports the average cost borne by households for institutional deliveries, care for children, and other exempted healthcare services. Independent of the covered service, the mean cost to households under the current free healthcare policy was US$11.76. Disaggregated figures showed that the mean costs to households were US$21.24, US$5.76 and US$13.33 for delivery, care for children, and other exempted services, respectively. The results show a significant reduction in household costs for the delivery, from US$70.90 (before the EmOC subsidy policy, i.e., user fee charged at the time) to US$21.24 under the free healthcare policy for maternal and child health. The mean household costs for delivery were US$21.78 and US$28.33 in the district and regional hospital facilities before the free healthcare policy and US$21.24 under the policy. Interestingly, the findings also show null median costs for delivery care, care for children and other exempted services, including prenatal care and postnatal.

1. **Discussion**

This study is the first attempt to investigate at a national level the effects of the free healthcare policy for maternal care and care of children under five years old in Burkina Faso. This work is unique since it comprehensively studied the policy's effects on nationwide service use and health outcomes. Most previous studies have limited scope and primarily assess free healthcare experiments ([12](#_ENREF_12), [19](#_ENREF_19), [24](#_ENREF_24)). In addition, this study critically interrogated the effects of the free healthcare policy for maternal and child health in light of the contextual situation of the country, integrating the effects of insecurity. None of the previous studies has attempted to take the effects of insecurity into account. Because of its scope, the present investigation brought several insights worthy of discussion.

**5.1 Effects on the use of services**

The findings show a significant increase in the use of healthcare facilities for consultations for children under five years old. This finding is consistent with previous research that found increased use of healthcare services by children following the introduction of the free healthcare policy for maternal and child health in Burkina ([12](#_ENREF_12), [25](#_ENREF_25), [26](#_ENREF_26)). The finding also aligns with numerous review papers demonstrating increased service use following user fee removal ([27-29](#_ENREF_27)). Though not significant at the 95% confidence level, the findings also show increased use of healthcare facilities for assisted deliveries, second antennal visits, complicated deliveries, and reduced cesarean sections. The increased use of health facilities for antenatal visits and assisted deliveries was consistent with findings from numerous previous studies. Research has shown that user fee removal is associated with increased utilization of health facilities for delivery in Sudan ([30](#_ENREF_30)) and some pilot experiments in Burkina Faso ([19](#_ENREF_19), [24](#_ENREF_24)). The apparent increase in antenatal consultations and assisted deliveries seems also to align with findings from [Hangoma, Robberstad (31)](#_ENREF_31), [Xu, Evans (32)](#_ENREF_32), and [John (30)](#_ENREF_30). Unlike a previous review finding that the abolition of user fees led to increased caesarian sections ([33](#_ENREF_33)), this study showed a reduction in caesarian sections. This divergent result could be explained by the increased utilization of health facilities for antenatal consultations and assisted deliveries. Antenatal consultations are critical for the early detection of abnormalities and signs of complications as well as proper management of the delivery. Increased antenatal consultations and assisted deliveries could be why more complicated deliveries were managed in hospital facilities. These, in turn, may well explain the reduction in caesarians that would have otherwise been performed to save lives.

**5.2 Effects on health outcomes**

The introduction of the policy significantly reduced mortality from severe malaria in children under the age of five. Though insignificant, the results also indicated a decrease in intrahospital infant mortality. The increased use of consultative care for children under five could mediate the reduced mortality from severe malaria in children under five. A substantial body of research has demonstrated the association between access to healthcare and reduced mortality in children under five years old ([22](#_ENREF_22), [34](#_ENREF_34), [35](#_ENREF_35)). In Burkina Faso, many researchers that have evaluated the impacts of the introduction of the free healthcare policy for maternal and child health reported increased use of healthcare services by children in rural ([25](#_ENREF_25)) and in both rural and urban areas ([12](#_ENREF_12), [26](#_ENREF_26)). Our findings align with a previous experiment that showed increased use of consultative care services for children under five years old in Kaya, Burkina Faso ([18](#_ENREF_18)). Increased use of health facilities for curative and preventive care after removing user fees has also been reported by several studies in many other places ([27](#_ENREF_27), [29](#_ENREF_29), [32](#_ENREF_32), [36](#_ENREF_36)), thereby indicating a significant contribution of the policy to the improvement of children’s health. Evidence of increased use of health services by children may well contribute to reducing deaths from severe malaria through promptly initiating appropriate care. Numerous other studies have shown that delivery in a health facility with a skilled provider reduces early neonatal and infant mortality ([37-39](#_ENREF_37)). This study also showed increased institutional deliveries, though not significantly. Several other studies conducted in Burkina Faso have demonstrated increased facility-based deliveries following the launching of the free healthcare policy for maternal and child health ([24](#_ENREF_24)). Increased hospital deliveries may have also contributed to reducing the overall child mortality, including mortality caused by severe malaria in children under five.

**5.3 Effects on household costs**

The findings show that the user fee removal policy has further reduced costs borne by households compared to cost levels before the introduction of the free health care policy. It has even eliminated delivery costs and care costs for children and other exempted healthcare services for half of the households. Previous studies conducted in Burkina Faso also demonstrated that the fee exemption policy, whether partial or not, was ineffective in eliminating costs as it was supposed to ([20](#_ENREF_20), [40](#_ENREF_40)). The user fee removal policy has only removed costs for half of the studied population, as shown by the median cost of 0. However, it still needs to remove all the direct costs related to delivery, child care and other exempted services for all households. This finding is remarkably consistent with a study in Burkina Faso which found that the user fee removal policy did not remove all the direct costs it was supposed to ([40](#_ENREF_40)). The finding is also in line with previous other research studies, which have found that the introduction of user fee removal policies has failed to remove household costs for care in Uganda ([32](#_ENREF_32)), Ghana ([41](#_ENREF_41)) and more recently in Zambia ([31](#_ENREF_31)).

1. **Limitations**

This study had several restraints. Though there was a gentle application of time-series techniques in this investigation, the absence of a control group may have weakened the power of the findings. Secondly, the study focused on measuring the effects of the user fee removal policy on the use of services and resulting outcomes using NHIS data. Though the country’s NHIS has often been acknowledged as appropriate ([12](#_ENREF_12), [24](#_ENREF_24)), it does not collect users’ socio-economic characteristics. Because of this, it was not possible to conduct a detailed analysis of the distributive impact of user fee removal as described by a previous study ([45](#_ENREF_45)). Third, several supply and demand side factors affecting health service use were controlled for, including health personnel density, distance, and insecurity. However, the likelihood that some critical factors affecting the use of health services, such as cultural factors or quality of services, may have yet to be accounted for since NHIS does not collect this data routinely. These also may have affected the study’s findings.

1. **Conclusions**

This paper contributes to the body of evidence on the cost, and effects of user fee removal policies on maternal and children’s health. The originality of this study lies in the fact that it evaluated the effect of the health policy reform at the country level, taking into account contextual factors such as insecurity. Insecurity has never been incorporated in previous evaluations.

The results suggest that user fee removal policies for maternal and children’s health increased consultations for children under five and reduced mortality from severe malaria in children under five. The findings also show that the policy eliminated healthcare costs for half of the people. There seem to be indications of increased service use for the other studied indicators. Given the positive effects, the findings of this investigation support the pursuit of its implementation.

**Abbreviations**

| BCEAO | West African Economic and Monetary Union Central Bank |
| --- | --- |
| CI | Confidence Interval |
| EMOC | Emergency Obstetric Care |
| FCFA | Franc de la Communauté Financière de l'Afrique |
| GDP | Gross Domestic Product |
| GNI | Gross National Income |
| IMF | International Monetary Fund |
| IRR | Incidence Rate Ratio |
| MoH | Ministry of Health |
| NHIS | National Health Information System |
| OLS | Ordinary Least Square |
| PNDES | National Plan for Economic and Social Development |
| UFR | User Fee Removal |
| US$ | United States Dollar |

1. **References**

1. Countdown to 2015. Maternal, Newborn and Child Survival: A Decade of Tracking Progress for Maternal, Newborn and Child Survival

2. Borghi J, Hanson K, Acquah C, Ekanmian G, Filippi V, Ronsmans C. Costs of near-miss obstetric complications for women and their families in Benin and Ghana. Health policy and planning. 2003;8(18):383–90.

3. Storeng K, Baggaley R, Ganaba R, Ouattara F, Akoum M, Filippi V. Paying the price: The cost and consequences of emergency obstetric care in Burkina Faso. Social science & medicine. 2008;66(3):545–57.

4. Ilboudo PG, Russell S, D'Exelle B. The long term economic impact of severe obstetric complications for women and their children in Burkina Faso. PloS one. 2013;8(11):e80010.

5. Asfaw A, Braun Jv, Klasen S. How Big is the Crowding-Out Effect of User Fees in the Rural Areas of Ethiopia? Implications for Equity and Resources Mobilization. World development. 2004;32(12):2065-81.

6. McKinnon B, Harper S, Kaufman JS, Bergevin Y. Removing user fees for facility-based delivery services: a difference-in-differences evaluation from ten sub-Saharan African countries. Health policy and planning. 2015;30(4):432-41.

7. Meessen B, Hercot D, Noirhomme M, Ridde V, Tibouti A, Tashobya CK, et al. Removing user fees in the health sector: a review of policy processes in six sub-Saharan African countries. Health policy and planning. 2011;26 Suppl 2:ii16-29.

8. Campbell O, Graham W. Strategies for reducing maternal mortality: getting on with what works. The Lancet Maternal Survival series. 2006;368(9543):1284-99.

9. Yates R. Universal health care and the removal of user fees. The Lancet. 2009;373:2078–81.

10. Ridde V, Morestin F. A scoping review of the literature on the abolition of user fees in health care services in Africa. Health policy and planning. 2011;26(1):1-11.

11. Ridde V, Queuille L, Atchessi N, Samb O, Heinmüller R, Haddad S. L’évaluation d’une expérimentation d’exemption du paiement des soins pour les groupes vulnérables au Burkina Faso. 2012.

12. Zombre D, De Allegri M, Ridde V. Immediate and sustained effects of user fee exemption on healthcare utilization among children under five in Burkina Faso: A controlled interrupted time-series analysis. Social science & medicine. 2017;179:27-35.

13. Kuunibe N, Lohmann J, Hillebrecht M, Nguyen HT, Tougri G, De Allegri M. What happens when performance-based financing meets free healthcare? Evidence from an interrupted time-series analysis. Health policy and planning. 2020;35(8):906-17.

14. INSD. Cinquième Recensement Général de la Population et de l’Habitation du Burkina Faso. Chiffres clés des résultats définitifs. 2022.

15. Ministère de la santé. Annuaire statistique 2021. Ouagadougou, Burkina Faso; 2021.

16. Global Health Observatory. Country views: Burkina Faso statistics summary (2002 - present) Geneva, Switzerland: WHO; 2019 [updated 2019-02-08. Available from: <http://apps.who.int/gho/data/node.country.country-BFA?lang=en>.

17. Ministère de la Santé. Arrêté conjoint n2018-1211 MS/MINEFID portant adoption du Manuel de procédures descriptives des modalités de gestion, de suivi et de contrôle des mesures de gratuité des soins au profit des femmes et des enfants de moins de cinq (05) ans vivant au Burkina Faso. Ouagadougou, Burkina Faso2018.

18. Druetz T, Fregonese F, Bado A, Millogo T, Kouanda S, Diabate S, et al. Abolishing Fees at Health Centers in the Context of Community Case Management of Malaria: What Effects on Treatment-Seeking Practices for Febrile Children in Rural Burkina Faso? PloS one. 2015;10(10):e0141306.

19. Johri M, Ridde V, Heinmuller R, Haddad S. Estimation of maternal and child mortality one year after user-fee elimination: an impact evaluation and modelling study in Burkina Faso. Bulletin of the World Health Organization. 2014;92(10):706-15.

20. Ganaba R, Ilboudo PG, Cresswell JA, Yaogo M, Diallo CO, Richard F, et al. The obstetric care subsidy policy in Burkina Faso: what are the effects after five years of implementation? Findings of a complex evaluation. BMC pregnancy and childbirth. 2016;16:84.

21. Anand S, Barnighausen T. Human resources and health outcomes: cross-country econometric study. Lancet. 2004;364(9445):1603-9.

22. Rutherford ME, Mulholland K, Hill PC. How access to health care relates to under-five mortality in sub-Saharan Africa: systematic review. Tropical medicine & international health : TM & IH. 2010;15(5):508-19.

23. United Nations. Treasury - UN Operational Rates of Exchange 2018 [Available from: https://treasury.un.org/operationalrates/OperationalRates.php.

24. Nguyen HT, Zombre D, Ridde V, De Allegri M. The impact of reducing and eliminating user fees on facility-based delivery: a controlled interrupted time series in Burkina Faso. Health policy and planning. 2018;33(8):948-56.

25. Zombre D, De Allegri M, Platt RW, Ridde V, Zinszer K. An Evaluation of Healthcare Use and Child Morbidity 4 Years After User Fee Removal in Rural Burkina Faso. Maternal and child health journal. 2019;23(6):777-86.

26. Debe S, Ilboudo PG, Kabore L, Zoungrana N, Gansane A, Ridde V, et al. Effects of the free healthcare policy on health services' usage by children under 5 years in Burkina Faso: a controlled interrupted time-series analysis. BMJ open. 2022;12(11):e058077.

27. Lagarde M, Palmer N. The impact of user fees on health service utilization in low- and middle-income countries: how strong is the evidence? Bulletin of the World Health Organization. 2008;86(11):839-48.

28. Lagarde M, Palmer N. The impact of user fees on access to health services in low- and middle-income countries. The Cochrane database of systematic reviews. 2011(4):Cd009094.

29. McPake B, Brikci N, Cometto G, Schmidt A, Araujo E. Removing user fees: learning from international experience to support the process. Health policy and planning. 2011;26(suppl_2):ii104-ii17.

30. John EU. The Impacts of User Fees on Health Services in Sub-Saharan African Countries: A Ctirical Analysis of the Evidence. American Journal of Public Health Research. 2013;1(8):196-202.

31. Hangoma P, Robberstad B, Aakvik A. Does Free Public Health Care Increase Utilization and Reduce Spending? Heterogeneity and Long Term Effects. World development. 2018;101:334-50.

32. Xu K, Evans DB, Kadama P, Nabyonga J, Ogwal PO, Nabukhonzo P, et al. Understanding the impact of eliminating user fees: utilization and catastrophic health expenditures in Uganda. Social science & medicine. 2006;62(4):866-76.

33. Hatt LE, Makinen M, Madhavan S, Conlon CM. Effects of user fee exemptions on the provision and use of maternal health services: a review of literature. Journal of health, population, and nutrition. 2013;31(4 Suppl 2):67-80.

34. Bhutta ZA, Das JK, Bahl R, Lawn JE, Salam RA, Paul VK, et al. Can available interventions end preventable deaths in mothers, newborn babies, and stillbirths, and at what cost? Lancet. 2014;384(9940):347-70.

35. World Health Organisation. Children: Reducing Mortality 2021 [

36. Masiye F, Chitah BM, McIntyre D. From targeted exemptions to user fee abolition in health care: experience from rural Zambia. Social science & medicine. 2010;71(4):743-50.

37. Goudar SS, Goco N, Somannavar MS, Vernekar SS, Mallapur AA, Moore JL, et al. Institutional deliveries and perinatal and neonatal mortality in Southern and Central India. Reproductive health. 2015;12 Suppl 2:S13.

38. Moyer CA, Dako-Gyeke P, Adanu RM. Facility-based delivery and maternal and early neonatal mortality in sub-Saharan Africa: a regional review of the literature. African journal of reproductive health. 2013;17(3):30-43.

39. Pattinson R, Kerber K, Buchmann E, Friberg IK, Belizan M, Lansky S, et al. Stillbirths: how can health systems deliver for mothers and babies? Lancet. 2011;377(9777):1610-23.

40. Meda IB, Baguiya A, Ridde V, Ouédraogo HG, Dumont A, Kouanda S. Out-of-pocket payments in the context of a free maternal health care policy in Burkina Faso: a national cross-sectional survey. Health economics review. 2019;9(1):11.

41. Asante F, Chikwama C, Daniels A, Armar-Klemesu M. Evaluating the economic outcomes of the policy of fee exemption for maternal delivery care in ghana. Ghana medical journal. 2007;41(3):110-7.

42. Bernal JL, Cummins S, Gasparrini A. Interrupted time series regression for the evaluation of public health interventions: a tutorial. International journal of epidemiology. 2017;46(1):348-55.

43. Lagarde M. How to do (or not to do) ... Assessing the impact of a policy change with routine longitudinal data. Health policy and planning. 2012;27(1):76-83.

44. Wagner AK, Soumerai SB, Zhang F, Ross-Degnan D. Segmented regression analysis of interrupted time series studies in medication use research. Journal of clinical pharmacy and therapeutics. 2002;27(4):299-309.

45. Chama-Chiliba MC, Koch FS. Assessing regional variations in the effect of the removal of user fees on institutional deliveries in rural Zambia. 2014.
